# Complete mitochondrial genome of Sri Lankan Junglefowl (*Gallus lafayetti*) and phylogenetic study

**DOI:** 10.1080/23802359.2017.1422409

**Published:** 2018-01-05

**Authors:** Nalini Yasoda Hirimuthugoda, Adeniyi C. Adeola, Xing Chen, Patthamesthrige Wimal Anthony Perera, Weligalle Wedarallage Dewar Asoka Gunawardena, Humpita Gamaralalage Thilini Nisanka Gunwardana, Ting-Ting Yin, Ming-Shan Wang, Gui-Mei Li, Min-Sheng Peng, Ya-Ping Zhang

**Affiliations:** a Department of Animal Science, Faculty of Agriculture, University of Ruhuna, Matara, Sri Lanka;; b Yunnan Laboratory of Molecular Biology of Domestic Animals, Kunming Institute of Zoology, Chinese Academy of Sciences, Kunming, China;; c Kunming College of Life Science, University of Chinese Academy of Sciences, Kunming, China;; d Kunming Biological Diversity Regional Center of Large Apparatus and Equipments, Kunming Institute of Zoology, Chinese Academy of Sciences, Kunming, China;; e State Key Laboratory for Conservation and Utilization of Bio-Resources, Yunnan University, Kunming, China

**Keywords:** Mitochondrial DNA, next-generation sequencing, phylogenetic analysis, Sri Lankan junglefowl

## Abstract

The complete mitochondrial genomes of two Sri Lankan junglefowl (*Gallus lafayetti*: CJF) individuals were sequenced by using next-generation sequencing technique. Samples were collected from Rathnapura and Pelmadulla areas in Sri Lanka. The complete mitochondrial DNA is 16,839 bp in length, with a typical mitogenome structure composed of a non-coding control region, 22 tRNA, two rRNA, and 13 protein-coding genes. Overall base composition is 30% A, 23.9% T, 32.3% C, and 13.6% G indicating high content of 54.0% A + T for both individuals. Phylogenetic analysis reveals that CJF samples cluster with the clade of the green junglefowl (*Gallus varius*) and red junglefowl (*Gallus gallus*) than to grey junglefowl (*Gallus sonerattii*: GyJF). This result can be subsequently used to provide essential information for junglefowl evolution.

## Introduction

The Sri Lankan junglefowl (*Gallus lafayetti*: CJF), also known as Ceylon junglefowl, is one of the species in genus *Gallus* among the four species (grey junglefowl (*G. sonneratii*; GyJF), green junglefowl (*G. varius*; GJF), red junglefowl (*G. gallus*; RJF) and CJF) (Delacour 1977; Sibley and Ahlquist 1990; Johnsgard 1999). It is endemic to Sri Lanka and considered as the national bird of Sri Lanka, distributed in Yala National Park in dry zone, Sinharaja rain forest and most of tea estates. Being geographically isolated in island from Indian sub-continent, very few genomic and evolutionary studies have been carried out on CJF to understand its phylogeny. The total genomic DNA of the two CJF individuals from Rathnapura and Pelmadulla areas in Sri Lanka was extracted from the whole blood with standard phenol/chloroform methods. The PCR, library construction, next-generation sequencing, and *de novo* assembly for mitochondrial DNA (mtDNA) genomes followed the previous protocol (Chen et al. 2016). Caveats were followed for quality control in mtDNA data analyses (Shi et al. 2014). The variants were scored and checked manually relative to the reference sequence AP003321 (Nishibori et al. 2005) and the bam file was exported by Torrent Suite 5.0.2 to confirm the scored variants by using Integrative Genomics Viewer (Thorvaldsdóttir et al. 2013).

We obtained the complete mtDNA genomes of two CJF individuals (GSA No. PRJCA000289 and PRJCA000290) in our study. We described 16,839 bp of CJF mitochondrial genomic sequences, including a non-coding control region, 22 transfer RNA (tRNA) genes, two ribosomal RNA (rRNA) genes, and 13 protein-coding genes. On the average, overall base composition of the mitochondrial genomes is as follows: 30.1% A, 23.9% T, 32.3% C, and 13.6% G, showing high content of 54.0% A + T for both samples. Nucleotide composition was estimated by MEGA 7.0 (Kumar et al. 2016). The three CJF sequences (two *de novo* and one from previous study, Nishibori et al. 2005) had 100% bootstrap support from neighbour-joining (NJ), maximum likelihood (ML), and maximum parsimony (MP) analyses. Therefore, this provides further evidence for the validity of the sequences obtained in our study.

**Figure 1.**
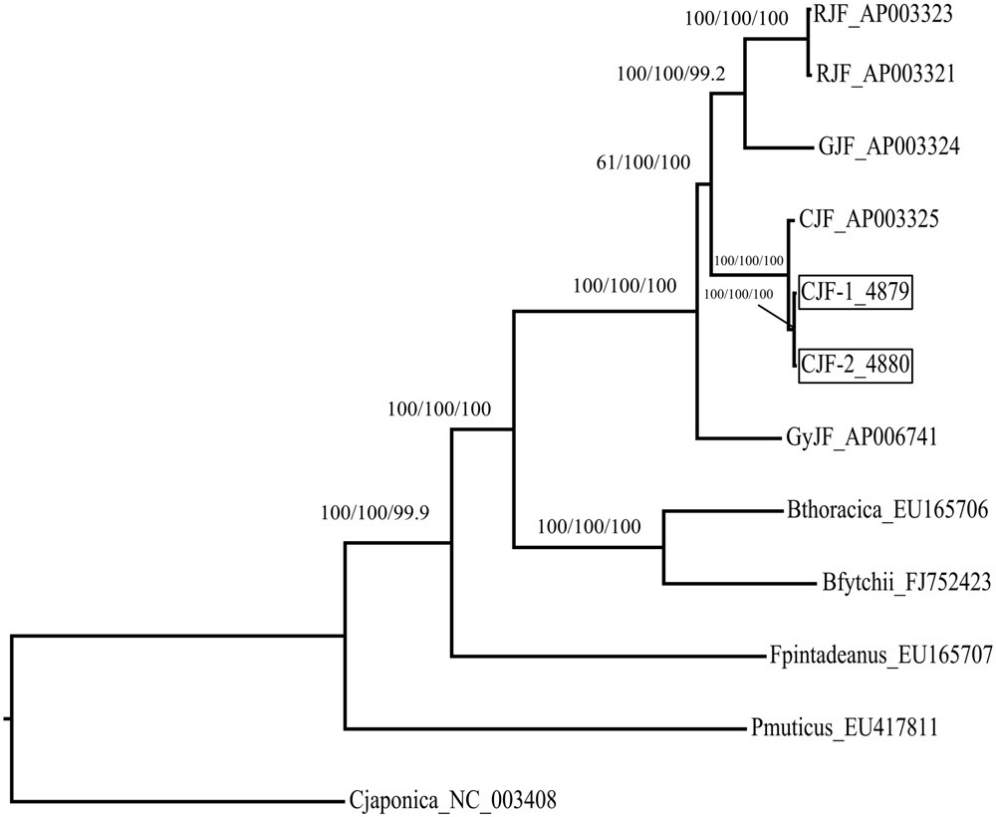
Phylogenetic tree of the relationships among Galliformes based on mtDNA genome sequences. *Pavo muticus* served as the outgroup. Numbers above each node indicates the NJ, ML, and MP bootstrap support values, respectively. All the species’ accession numbers are listed as follows: *Gallus gallus* (RJF) AP003321, AP003323; *G. varius* (GJF) AP003324; *G. sonneratii* (GyJF) AP006741; *G. lafayettei* (CJF) AP003325, PRJCA000289–4880, PRJCA000290–4879; *Bambusicola thoracicus* (Bthoracica) EU165706; *B. fytchii* (Bfytchii) FJ752423; *Coturnix japonica* (Cjaponica) NC_003408; *Francolinus pintadeanus* (Fpintadeanus) EU165707; *Pavo muticus* (Pmuticus) EU417811.

The phylogenetic position of CJF was estimated from complete mtDNA sequences. Neighbour-joining analyses were performed in MEGA 7.0, ML calculated in RaxML (Randomized A(x)ccelerated Maximum Likelihood) (Stamatakis et al. 2008; https://embnet.vital-it.ch/raxml-bb/), and MP estimated using PAUP* (V4.0) (Swofford 2003). The phylogenetic analysis results show that the *Gallus* four species clustered in three different clades. RJF and GJF grouped in closer clades while CJF is more distance from GyJF. This is in contrast with Helm-Bychowski and Wilson (1986) and Fumihito et al. (1996), our findings showed that having a common ancestor CJF is more closely related to both GJF and RJF than to GyJF which is in accordance with Guan et al. (2009). This study will contribute to the phylogenetic analyses in Galliformes (Figure 1).
